# On the importance of interpretable machine learning predictions to inform clinical decision making in oncology

**DOI:** 10.3389/fonc.2023.1129380

**Published:** 2023-02-28

**Authors:** Sheng-Chieh Lu, Christine L. Swisher, Caroline Chung, David Jaffray, Chris Sidey-Gibbons

**Affiliations:** ^1^ Section of Patient-Centered Analytics, Division of Internal Medicine, The University of Texas MD Anderson Cancer Center, Houston, TX, United States; ^2^ The Ronin Project, San Mateo, CA, United States; ^3^ The Lawrence J. Ellison Institute for Transformative Medicine, Los Angeles, CA, United States; ^4^ Department of Radiation Oncology, The University of Texas MD Anderson Cancer Center, Houston, TX, United States; ^5^ Institute for Data Science in Oncology, The University of Texas MD Anderson Cancer Center, Houston, TX, United States; ^6^ Department of Imaging Physics, The University of Texas MD Anderson Cancer Center, Houston, TX, United States; ^7^ Department of Radiation Physics, The University of Texas MD Anderson Cancer Center, Houston, TX, United States

**Keywords:** opaque machine learning models, interpretability and explainability, decision-making support, high-stakes prediction, precision medicine

## Abstract

Machine learning-based tools are capable of guiding individualized clinical management and decision-making by providing predictions of a patient’s future health state. Through their ability to model complex nonlinear relationships, ML algorithms can often outperform traditional statistical prediction approaches, but the use of nonlinear functions can mean that ML techniques may also be less interpretable than traditional statistical methodologies. While there are benefits of intrinsic interpretability, many model-agnostic approaches now exist and can provide insight into the way in which ML systems make decisions. In this paper, we describe how different algorithms can be interpreted and introduce some techniques for interpreting complex nonlinear algorithms.

## 1 Introduction

Machine learning (ML) techniques have demonstrated exceptional promise in producing reliable predictions to inspire action across diverse industries. They have been fundamental in the automation of complex tasks such as language translation, self-driving vehicles, as well as internet search and recommendation engines. In oncology, there are many applications across the care continuum from informing healthcare policy, managing clinical operations, to providing individualized insights into direct patient care (1–4).

The principle of using data-driven prediction models to inform clinical oncology care is not new, though the increased availability and maturity of the capabilities of ML techniques has led to renewed interest in the topic. Traditional prediction tools tend to be developed using statistical methodologies (5). For example, oncology nomograms often utilized linear algorithms to create tools with which future outcomes could be predicted. These models were often based on ordinal least squares regression techniques which offered straightforward interpretability using coefficients. However, machine learning’s ability to characterize nonlinear interactions between features has led to potential issues with understanding the relationship between the input features and the output prediction. These nonlinear algorithms are often referred to as ‘black boxes’ which may produce accurate predictions but at the expense of clear and concise interpretability. Although many ML models can be adequately thought of as ‘black boxes’, it is not true that all ML algorithms are uninterpretable. In previous work (6, 7), we have previously described a continuum of algorithms ranging from ‘Auditable Algorithms’ to ‘Black Boxes’ and argued that interpretability necessarily became more difficult and the ability to estimate highly complex nonlinear models increased.

In recent years, the machine learning community has produced several significant advancements into providing some level of interpretability for complex nonlinear algorithms (8, 9). Explainability and interpretability are two tied concepts (10). There are no clear and widely-accepted definitions of these terms, so we will use a working definition inspired by other sources (11). Explainability refers to the ability to describe the elements of an ML model, which might include the provenance and nature of the training data, weights, and coefficients of the models, or the importance of different features in deriving the prediction (10). Explainability asks the question “can we *describe* the different elements of the model?”. The concept of interpretability goes beyond that of description of explainability and asks “can we *understand* the reasoning behind the model’s prediction?” (11, 12). In this paper, we will focus on the description of features and explanation approaches that make an ML model interpretable by allowing humans to gain insight into model reasoning and consistently predict model outputs.

Interpretability is an important concept within clinical ML as model performance is unlikely to be perfect, and the provision of an interpretable explanation can aid in decision-making using ML models. The importance of interpretability for all ML-based decision-making algorithms is demonstrated in the United States Government’s Blueprint for an AI Bill of Rights which introduces “Notice and Explanation” as a key principle for ML-based prediction models (13). Additionally, the U.S. Food and Drug Administration (FDA) guidelines for clinical decision support systems (CDSS) highlight the importance of providing the basis of predictions (14), and other regulatory and standards in healthcare and other industries (15).

For oncology practice, ML-based tools are often developed and used to support high-stakes decisions, such as diagnosis (16, 17), advance care planning communication (18, 19), and treatment selection (20). Providing only predictions is not enough to solve all problems for these tasks, and a model should provide explanations concerning its decision-making to allow human reasoning and preventative actions (11). Furthermore, interpretability is essential to ensure safety, ethics, and accountability of the models for ML models supporting oncology decisions (11). Inaccurate or biased predictions generated by a ML model can result in unintentional harms on both patients and institutions. In such cases, an explanation in model decision-making process pertaining to erroneous or discriminative predictions enables model auditing, debugging, and refinement to ensure model performance and fairness (11, 21).

Models making predictions using different types of data should be interpreted by different approaches. For instance, a common approach to interpret ML models leveraging image data is the salience map highlighting a portion of an image that is most relevant to model decisions (22). Many explanation approaches, such as attention, are also available for the provision of insights into the decision-making processes of models leveraging unstructured text data using natural language processing (23). As there is increasing enthusiasm for leveraging electronic health record data to construct predictive decision-making tools (19, 24, 25), we focus this paper on approaches most useful in deconstructing decision-making processes of opaque ML models using tabular data. Nevertheless, many of the interpretation approaches we covered are not data type constrained (23, 26).

In this manuscript, we demonstrate how interpretability and explainability of machine learning models can be informed both by algorithm selection and the applications of so-called “model agnostic” methods at the population and individual levels (8, 9). We also describe several of the benefits and limitations of both intrinsic interpretability such as that provided with logistic regression versus model agnostic methods. Additionally, we argue that interpretability can go beyond the drivers of an individual prediction and may also encompass methods to understand the quality, relevance, and distributions of training, testing, and inference data features which we used to inform the model. The intention of this manuscript was not to provide an exhaustive summary of state-of-the-art ML interpretation approaches but to introduce the concept of ML interpretability and explainability with practical examples to raise awareness of the topic among the oncology research community. For enthusiastic readers, there are systematic reviews that provide more compressive summaries of the existing model interpretation techniques (22, 27, 28).

## 2 Example models used to illustrate explanation methods for interpretability

In this paper, we demonstrate all model interpretation approaches with example models we created to identify cancerous breast masses using regularized linear regression (GLM), multivariate adaptive regression splines (MARS), k-Nearest Neighbors, Decision trees, extreme gradient boosting (XGB), and neural networks (NNET). We used the Breast Cancer Wisconsin Diagnostic Data Set which is publicly available from the University of California Irvine (UCI) ML Repository to train the models (29). There are 698 instances in the dataset with 9 categorical features (predictors). The features represented the characteristics of cell nuclei from breast masses sampled using fine-needle aspiration (FNA) (30). Possible values of each feature are 1 to 10, with 1 representing the closest to benign and 10 representing the closest to malignant. The outcome is a binary variable, which can either be benign or malignant. For simplicity, the dataset we used is relatively low dimensional, containing only 9 features, compared to most oncology research utilizing complex data with much more variables. Nevertheless, the interpretation approaches we discussed can be applied to models trained with high-dimensional data to provide rich insights beyond classification or regression outputs. Researchers have applied these methods to derive individualized, patient-centered information supporting clinical decision-making (31, 32) and to uncover disease risk/protective factors from their prognostic or diagnostic models trained with complex, high-dimensional datasets (33, 34).

We randomly split the dataset into a training set with 70% of the data for model development and a testing set with 30% of the data for model validation. For consistency and ease of reproducibility, we created our models with default configurations of the CARET (Classification And REgression Training) package without further hyperparameter optimization. We performed all modeling and analyses using the R statistical programming environment (version 4.2.1) (35) using CARET (version 6.0-93) (36), DALEX (version 2.4.2) (37), and lime (version 0.5.3) (38) packages. Reproducible code is available in the online supplement for all analyses.

## 3 Machine learning model interpretation approaches

Over the past decades since ML has been available, several approaches addressing interpretability issues of the ML-based models have been proposed and implemented (22). Some algorithms are interpretable-by-nature such as regularized logistic regression, nearest neighbors, and decision tree algorithms (26, 39, 40). We refer to these models as interpretable and refer to the interpretation methods as being model-specific. However, it can be practically impossible to comprehensively explain model outputs for models which rely on complete nonlinear data transformations, such as support vector machine and artificial neural networks, without applying model-agnostic approaches.

Moreover, model-specific approaches involve an understanding of the mechanism of the algorithm, whereas Linear Models, Decision Trees, etc. produce different explanations as results are highly impacted by feature selection and training hyperparameters. Interpretability in model-specific approaches is also undermined by feature complexity. Complex features (e.g., PCA-derived features), sparsity, lack of independence, monotonicity, and linearity do not guarantee interpretability. Finally, in some scenarios, particularly in larger datasets, simplicity may require sacrificing performance.

Model-agnostic approaches are a set of model interpretation methods that are applicable to ML models developed using any algorithms, including interpretable models. These methods can provide visualizations of model decision-making processes for human interpretation to answer questions, such as what the most important feature is for any model. The model-agnostic approaches can be further grouped into two categories, global and local interpretations. Global interpretations target uncovering average model decision-making processes at a dataset or cohort level, while local interpretations provide interpretations of model behaviors for individual predictions. Model agnostic approaches allow for flexibility in model choice, which means that there are more options to improve certain issues that may occur to a model in production that would necessitate the adoption of another algorithmic approach; a critical component to Safe and Effective ML Systems (13) and the FDA’s Good Machine Learning Practices GMLP (41).

**Figure 1:**
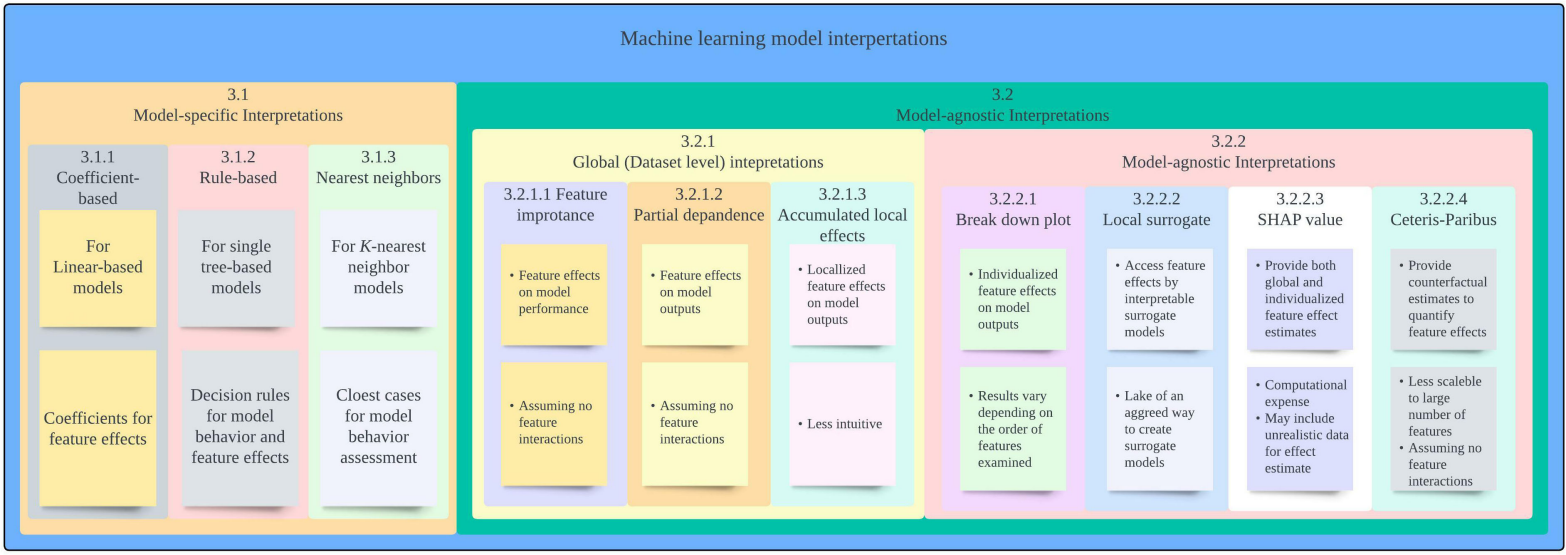
Summary of interpretation approaches covered. SHAP: Shapley additive explanation.

In the following section, we provide overview of each of the interpretation approaches along with an example showcasing the approach and its limitations. A summary of interpretation approaches covered in this paper is provided in **Figure 1**.

### 3.1 Model-specific interpretation approaches

#### 3.1.1 Coefficient-based method

Model-specific approaches refer to model interpretation methods that are available as an inherent part of certain ML algorithms (26). One of the most widely known and accessible approaches for model interpretation is assessing coefficients that are available for many linear models. By investigating the coefficient of each feature included in the prediction, we can know which features were used by a model to make predictions and how each variable contributes to the model output. As an example, the coefficients of our GLM model for breast mass cancerous predictions are presented in **Table 1**. The coefficients indicate that all features were included in the model were positively associated with a prediction of malignancy.

**Table 1:** Coefficients for the generalized linear model (GLM) with regularization.

Features	Coefficient
(Intercept)	-7.42
Normal Mitoses	0.70
Bland Chromatin	0.43
Adhesion	0.24
Cell Shape	0.23
Bare Nuclei	0.23
Cell Size	0.19
Epithelial Size	0.18
Normal Nucleoli	0.15
Thickness	0.06

Models adopting the MARS algorithm can be interpreted using a similar way. The MARS algorithm can be understood as an extension of a linear or rigid logistic regression model, which facilitates interactions between features whilst providing clear interpretability and deeper insight into the relationships in data (42, 43). Thus, the same coefficient method for interpreting a GLM model can be applied to interpret a MARS model. **Table 2** shows selected terms and their coefficients generated by our MARS model. We can use the information to calculate the probability of a breast mass sample being malignant and, in so doing, simulate the model behavior. The model identified interesting insights by revealing complex Thickness – Cell Size, Cell Size – Bare Nuclei, and Epithelial Size – Bare Nuclei interactions, indicating various effects on model outputs depending on feature values. Clinical implications of the feature interactions identified may not be obvious in our example, but it becomes explicit and important if our model predicts Hemoglobin A1c (HbA1c) using age groups and Body Mass Index (BMI). Strengths of associations between BMI and HbA1c varies among different age groups, suggesting that different glycemic control strategies should be used for different age populations (44).

**Table 2:** Features and coefficients determined by the model using the multivariate adaptive regression splines (MARS) algorithm.

No.	Feature	Coefficient
1	(Intercept)	1.09
2	h(Cell_Size-2)	-0.32
3	h(2-Cell_Size)	-0.75
4	h(Cell_Size-3)	0.33
5	h(Bare_Nuclei-2)	-0.37
6	h(Bare_Nuclei-3)	0.42
7	h(Thickness-5)×h(Cell_Size-2)	0.01
8	h(5-Thickness)×h(Cell_Size-2)	0.02
9	h(Cell_Size-3)×h(2-Bare_Nuclei)	0.99
10	h(3-Cell_Size)×h(2-Bare_Nuclei)	-0.17
11	h(Cell_Size-2)×h(2-Bare_Nuclei)	-0.83
12	h(2-Epithelial_Size)×h(Bare_Nuclei-2)	0.21

h(Variable – Constant) are hinge functions representing knots the multivariate adaptive regression splines model identified to better fit the data. The results of the functions are the maximum of 0 and the difference between the variable and constant values. For instance, suppose Cell_Size is 3, then h(Cell_Size-2)=Max(0, 3-2)=1.

Coefficients provide intuitive model interpretations to reveal model decision-making processes and enable easy implementation. Nevertheless, the approach provides less insight into the feature’s effect at the individual level. Fixed coefficients revealed by a model may not reflect the variances in features’ effects on model outputs among individuals. Further, due to the use of regularization methods, there is a possibility that GLM and MARS models drop features that are clinically considered to be important and associated with outcomes the models predict (45).

#### 3.1.2 Rule-based decision tree method

**Figure 2:**
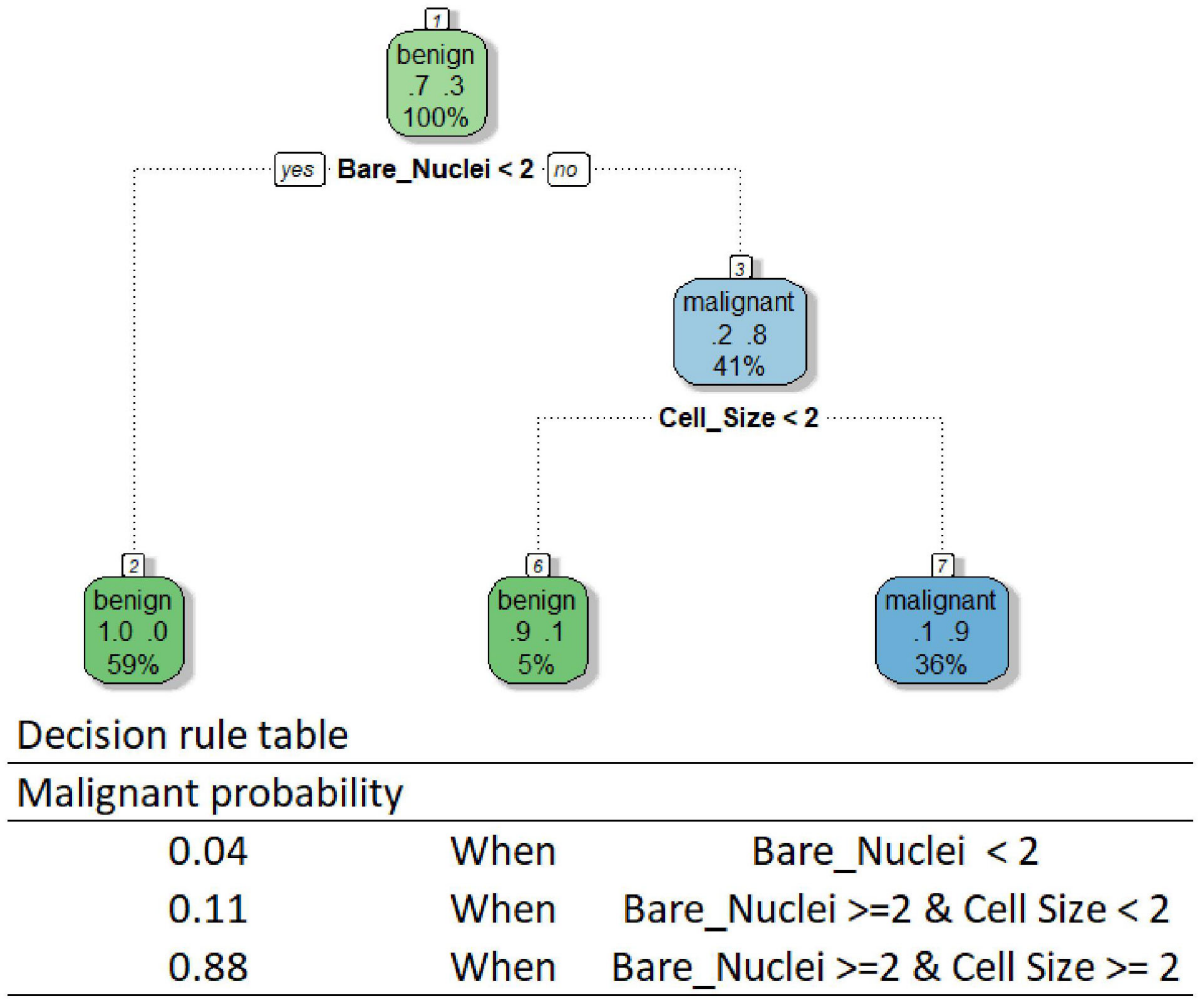
Decision tree (DT) and rule table.

Another widely recognized algorithm category is tree-based algorithms, which also allow intuitive interpretation whilst facilitating feature interactions (40). As their name suggests, these algorithms create a model by constructing a decision tree composed of a series of rules portioning data to determine model predictions. Although researchers have developed various tree-based algorithms, the method interpreting all models using these algorithms is the same if a single tree is developed (26). One can follow the rules of a tree-based model to reveal its decision-making process. We provided the decision tree and rule table used by our DT model as an example in **Figure 2**. The tree is relatively small in our case, while one can get a huge tree containing hundreds of branches for a complex predicting task using a high-dimensional dataset. According to the rules, our DT model makes predictions using only Bare Nuclei and Cell Size variables. Our model classifies a breast mass as malignant only when the Bare Nuclei and Cell Size scores of the mass are greater than or equal to 2.

Although the interpretation allows an easy understanding of the behavior of a tree-based model, the method is limited to models based on a single tree. The method becomes less useful for the interpretation of models using many powerful tree-based algorithms, such as the random forest algorithm, due to the creation of multiple trees for making predictions. Although we can draw all the trees and go through each tree to understand how the models behave, it is impossible to know what key features are used by these models to drive decisions and how the features influence the decisions by using this approach.

#### 3.1.3 Interpretation method for K-nearest neighbor models

A special class of ML models that allow interpretation without additional approaches are models using the *k*NN algorithm. A *k*NN model makes a prediction for a particular instance based on the neighbors of the instance (46). When predicting, a *k*NN model first identifies k instances most similar to the instance we are predicting from the training sample. Then, for a classification outcome, the model takes the most common class of the nearest neighbors identified. For continuous outcomes, the model averages the outcomes of the neighbors. Therefore, we can investigate the neighbors to understand the decision-making process of the model. For instance, we randomly selected a mass sample A from our validation sample and calculated the distance between features of A and all other masses in the training sample using the Euclidean distance method. As our KNN model used 7 nearest neighbors to determine predictions, the top seven instances with the smallest distances to A were the neighbors used by the model (**Table 3**). As four out of the seven neighbors were benign, our *k*NN model predicted that A was not a cancerous mass. The Euclidean distance between the seven neighbors and the instance A were 4.5 ± 0.7, while the distance between all training data and the instance A were 10.2 ± 2.7.

**Table 3:** Nearest neighbors of the example instance used by the k-Nearest Neighbor (KNN) model for prediction.

	Thickness	Cell size	Cell shape	Adhesion	Epithelial size	Bare nuclei	Bland Chromatin	Normal Nucleoli	Normal mitoses	Class	*d*
Predicting instance (A)	8	4	5	1	2	1	7	3	1	–	–
Neighbor 1	9	5	5	2	2	2	5	1	1	malignant	3.5
Neighbor 2	8	4	6	3	3	1	4	3	1	benign	3.9
Neighbor 3	10	4	3	1	3	3	6	5	2	malignant	4.4
Neighbor 4	6	3	3	3	3	2	6	1	1	benign	4.5
Neighbor 5	8	3	3	1	2	2	3	2	1	benign	4.8
Neighbor 6	6	2	1	1	1	1	7	1	1	benign	5.4
Neighbor 7	10	4	5	4	3	5	7	3	1	malignant	5.5

d: Euclidean distance to the predicting instance A. - means Not Applicable.

Through observing the features of the nearest neighbors selected for predictions, we can uncover on what basis our models make predictions. This approach is like case-based rationale which we often adopt to make decisions in our daily life by looking at the cases/conditions similar to our current encounters from our past experiences. It also offers opportunities for examining whether the cohort of the current case was represented in the creation of the kNN model. However, the interpretation method delivers no information about whether a feature is weighted over other features. Further, the approach does not uncover whether a feature is positively or negatively associated with the outcome.

Beyond the interpretations of simple algorithms, researchers have spent substantial efforts to develop model-specific interpretation approaches for models using complex models that are generally considered as not interpretable-by-nature, such as random forest (47), support vector machine (48), and neural networks (49, 50). Although these model-specific interpretation methods allow an easy understanding of model behaviors, the primary limitation is the limited flexibility of these methods. Use of these interpretation does not allow easy comparison among models using different algorithms Therefore, additional tools are needed to understand model decision-making processes when we pursue a higher-performing model with a more sophisticated algorithm.

### 3.2 Model-agnostic approaches

Model-agnostic interpretation approaches, contrary to model-specific methods, offer greater flexibility and can be applied to ML models using any algorithms. With flexibility, researchers can select any algorithms they believe are the best solution for solving the questions at hand and examine their models with consistent approaches for better model comparisons. These approaches use *post hoc* interpretation methods decomposing trained ML models (22). The general idea of the approach is to reveal model behaviors by observing the changes in model predictions when manipulating the input data instead of breaking down the models for an understanding of model structures. In the following section, we provided a gentle introduction to a few widely used approaches covering both global and local model-agnostic interpretation methods. Enthusiastic readers can find introductions to other model-agonistic methods in Molnar’s book addressing interpretability issues of ML (26).

#### 3.2.1 Global interpretation

The global interpretation methods focus on providing an overall picture depicting model behaviors at a dataset level. The approaches can help to reveal the averaged effect of a feature on model predictions for a given dataset. Of the global interpretation methods developed, feature importance (FI), partial dependence plots (PDP), and accumulated local effects (ALE) were the most widely used approaches in literature. We provide a description of these methods and demonstrate their utilization with our breast cancerous prediction model using the extreme gradient boosting trees algorithm (XGBT model).

##### 3.2.1.1 Feature importance

**Figure 3:**
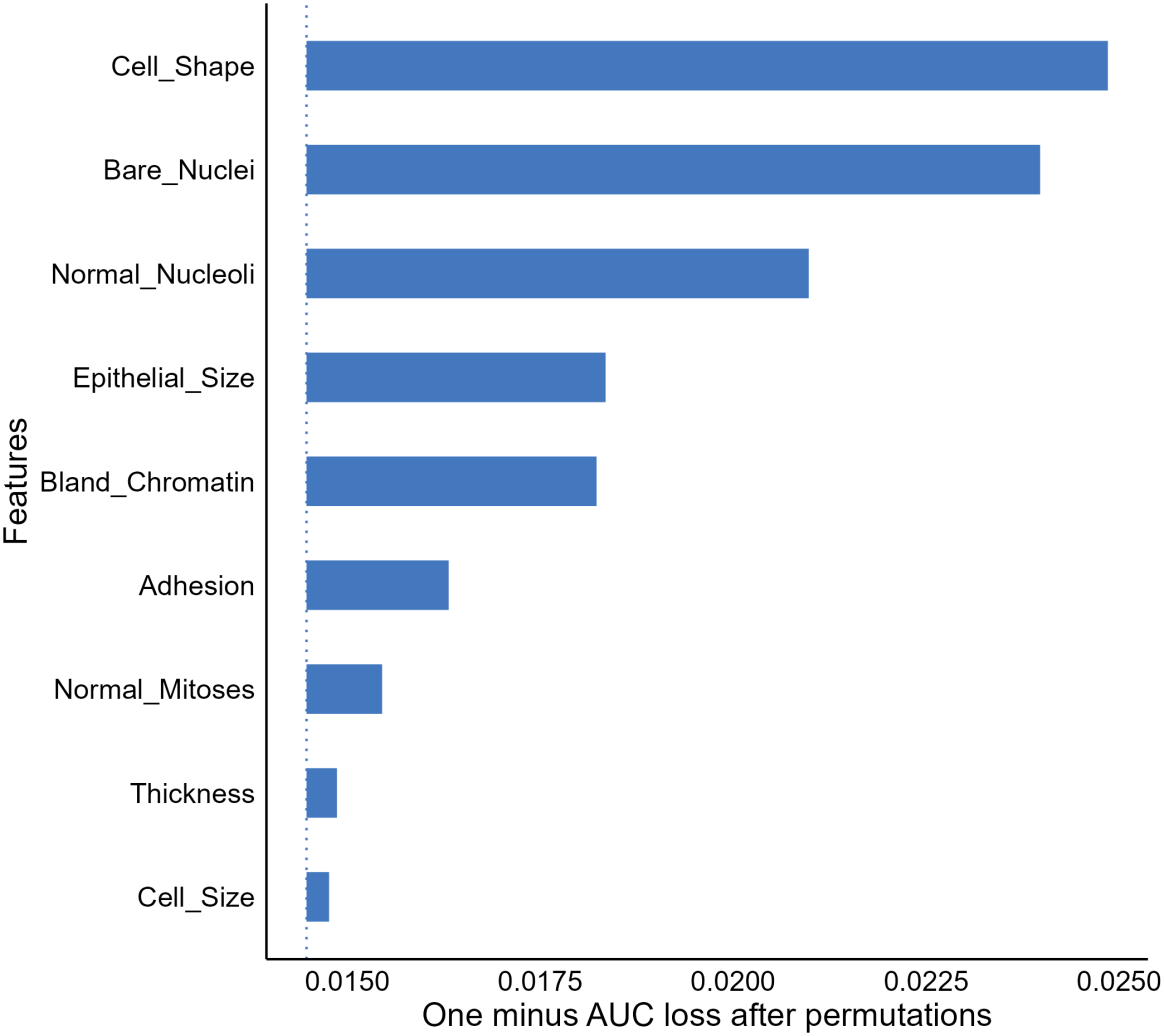
Feature importance (FI) analysis for the extreme gradient boosting tree model. AUC, Area under the receiver-operating characteristics.

A frequent question for a given predictive model beyond model performance is what features are important to the model for accurate predictions, which can be addressed by the FI analysis (51). The FI analysis estimates the importance of a feature by calculating model performance changes (e.g., loss in area under the receiver operating characteristic curve) when we randomly alter the feature’s value (52). A feature is deemed important if the performance loss is notable when permutating the feature’s value. Taking our XGBT model as an example, the FI analysis shows that cell shape, bare nuclei, normal mitoses, and epithelial size scores were the most important features enabling the model to generate accurate outputs (**Figure 3**).

The FI analysis is widely recognized as a useful approach allowing provision of compressed insights into model behaviors and is commonly utilized in medical ML literature (19, 53, 54). However, we should note that the result of the analysis does not reveal how features affect model decisions (52, 53). For instance, the FI result delivered no information on whether our XGBT model assigns a greater cancerous probability to a sample with a higher value in bland chromatin. In addition, due to the use of random permutation and intrinsic machine-selection of features, correlations between features can be problematic and result in unreliable feature importance estimates.

##### 3.2.1.2 Partial dependence plot

**Figure 4:**
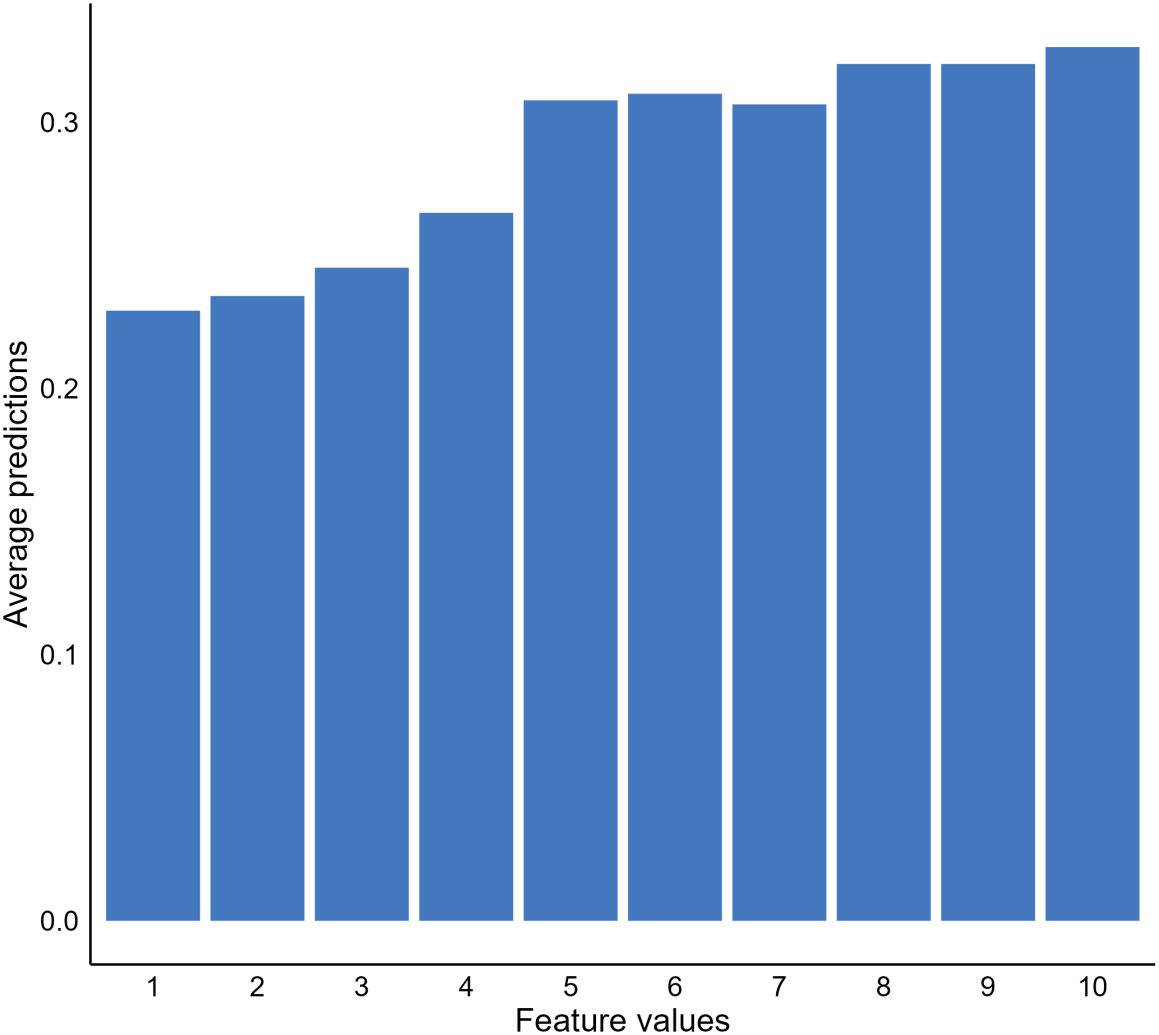
Relationship between bare nuclei score and predicted cancerous probability generated by our extreme gradient boosting tree (XGBT) model using partial dependence analysis.

In addition to important features, we may also be interested in knowing how the values of important features affect model predictions. A popular approach to address this question is to use partial dependence plots (PDP) to visualize the relationship between the outcome and a predicting feature of interest (22). The idea of the method is to estimate the relationships by marginalizing the feature of interest and calculating its marginal distribution (55, 56). For instance, suppose we want to know the relationship between the bare nuclei score of a breast mass sample and the predictions generated by our XGBT model; we can fix the value of the feature for all instances in the validation dataset and calculate a mean predicted malignancy probability. Next, we calculate the mean predicted probabilities for all possible values of the bare nuclei score (1–10) to uncover the probability distribution (marginal distribution). Through plotting out the probability distribution, we observe a positive relationship between the feature and the predicted malignancy probability generated by the model (**Figure 4**).

Although PDPs provide useful and intuitive model behavior interpretation, there are disadvantages of the approach that are important to highlight. First, the method assumes no interaction between features, which is not likely to be the truth for a real-world clinical dataset (26, 57). The approach can estimate feature effects based on unrealistic data. It is not apparent with our breast mass cancerous example. However, if our model was to predict house prices using room numbers and surface space, the approach could generate unrealistic data, such as ten rooms within a 100 square feet house. In such cases, the approach is not useful and can generate misleading results. Another limitation of PDPs was the use of a mean predicted probability and disregarding the distribution of the predicted probabilities when estimating the probabilities of the interested feature fixed with a certain value. The PDP results become less meaningful or even misleading if the distribution is scattered (26).

##### 3.2.1.3 Accumulated local effect

Another popular global model interpretation method is ALE, which addresses the same question as PDP does, while ALE provides more reliable model behavior information when correlations between features exist (57). The primary difference between the approaches is that ALE performs marginalization locally within instances with a similar value of the feature we are examining to avoid the use of unrealistic data for estimating model behaviors. Further, ALE uses differences in predicted probabilities generated for instances with similar values for the feature of interest as an alternative of mean to avoid the issue of a scattered distribution (57).

**Figure 5:**
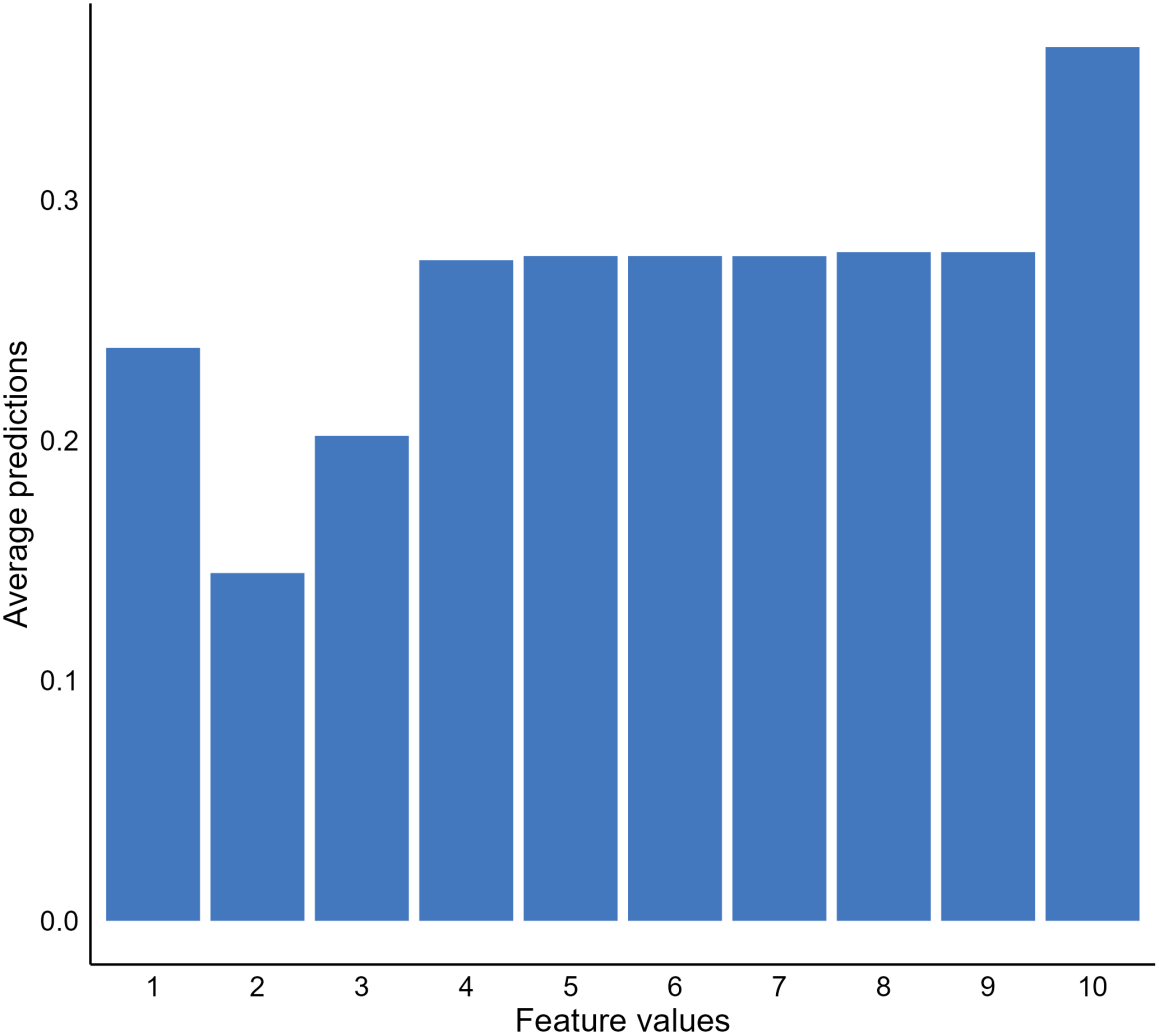
Accumulated local effect (ALE) analysis.

We again examined the effect of the bare nuclei score on the outputs of our XGBT model but using ALE. The ALE graph indicated the predicted probabilities notably increased when the bare nuclei score was ten, while the probabilities reduced when the feature was scored two (**Figure 5**). All other possible values of the feature resulted in similar predictions. The result is remarkably different from the PDP result. As correlations were likely to exist among the features in the breast mass dataset, we argued that the ALE provides more reliable information regarding the impact of specific features on model behavior.

The approach is not without limitations. The results of ALE are more complex compared to PDP and less interpretable, especially when strong and complex correlations between features exist (58). The ALE can still generate unstable interpretations of feature effects due to the arbitrary selection of numbers of intervals where local feature effects are estimated (11). Further, as ALE estimates feature effects per interval, the interpretation is interval specific and may not be applicable to other intervals (26). Nevertheless, the approach provides visual, unbiased interpretation of feature effects on model predictions and is recommended for interpreting models trained with clinical data that often involve correlated features (26).

#### 3.2.2 Local interpretation

Thus far, we have introduced several methods to uncover general model behaviors at the dataset level. As the primary utilizations of ML models are to provide individualized predictions, we may be interested in how a model makes predictions for individuals based on their data. Local interpretation methods were developed to uncover how much the value of each feature of an individual contributes to the ML model output for the individual to provide additional insights enabling individualized care (22, 27). In this section, we cover commonly used local interpretation approaches following the same structure we used in the previous section for global interpretations. To demonstrate the methods, we randomly selected an instance from the validation sample and examined the prediction generated by our NNET model for this instance. We provide the characteristics of the mass sample selected in **Table 4**.

**Table 4:** Feature of the mass sample selected.

Feature	Value
Thickness	8
Cell size	4
Cell shape	5
Adhesion	1
Epithelial size	2
Bare nuclei	1
Bland Chromatin	7
Normal Nucleoli	3
Normal mitoses	1
Class	Malignant

##### 3.2.2.1 Break down plot

**Figure 6:**
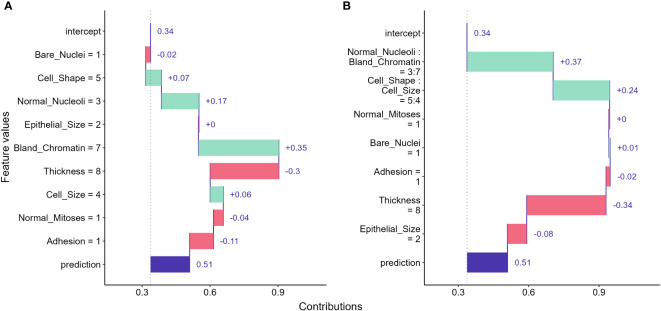
Break down (BD) plots showing how the contributions attributed to individual features for the instance we selected. **(A)** break down plot assuming no interactions among features; **(B)** break down plot with feature interactions considered.

One of the most straightforward approaches to examine the feature contributions to individual predictions is using a Break Down (BP) plot. The approach decomposes a model prediction into contributions and it then estimates the attribution of the contributions from each feature (22, 58). The intuition of the interpretation is to estimate the mean predictions for each feature when we consecutively fix an exploratory feature and permutate all other features (59). For instance, to examine the attribution for the sample we selected, we first computed the mean prediction by fixing the bare nuclei score to 1 and permutating all other features. As shown in **Figure 6A**, we got a mean prediction lower than the prediction for an intercept model by 0.02, indicating that having a bare nuclei score of 1 lowers the cancerous probability for the selected sample. For the next feature, we fix both the bare nuclei and cell shape score to calculate the mean prediction change. The process continued until all feature values were fixed.

Break down plots provide clear visualizations for us to evaluate feature contributions to individual predictions made by ML models. However, the disadvantage of the approach is that the order in which we examine features can significantly alters the result. The approach can provide misleading interpretations if feature interactions exist and the order is not carefully determined (58). When interactions between features exist, the interaction version of BD plot should be considered and may provide better information to address the ordering issue (**Figure 6B**). However, the BD plots for interactions can be computationally expensive and hard to understand for large feature numbers (58).

##### 3.2.2.2 Local surrogate

**Figure 7:**
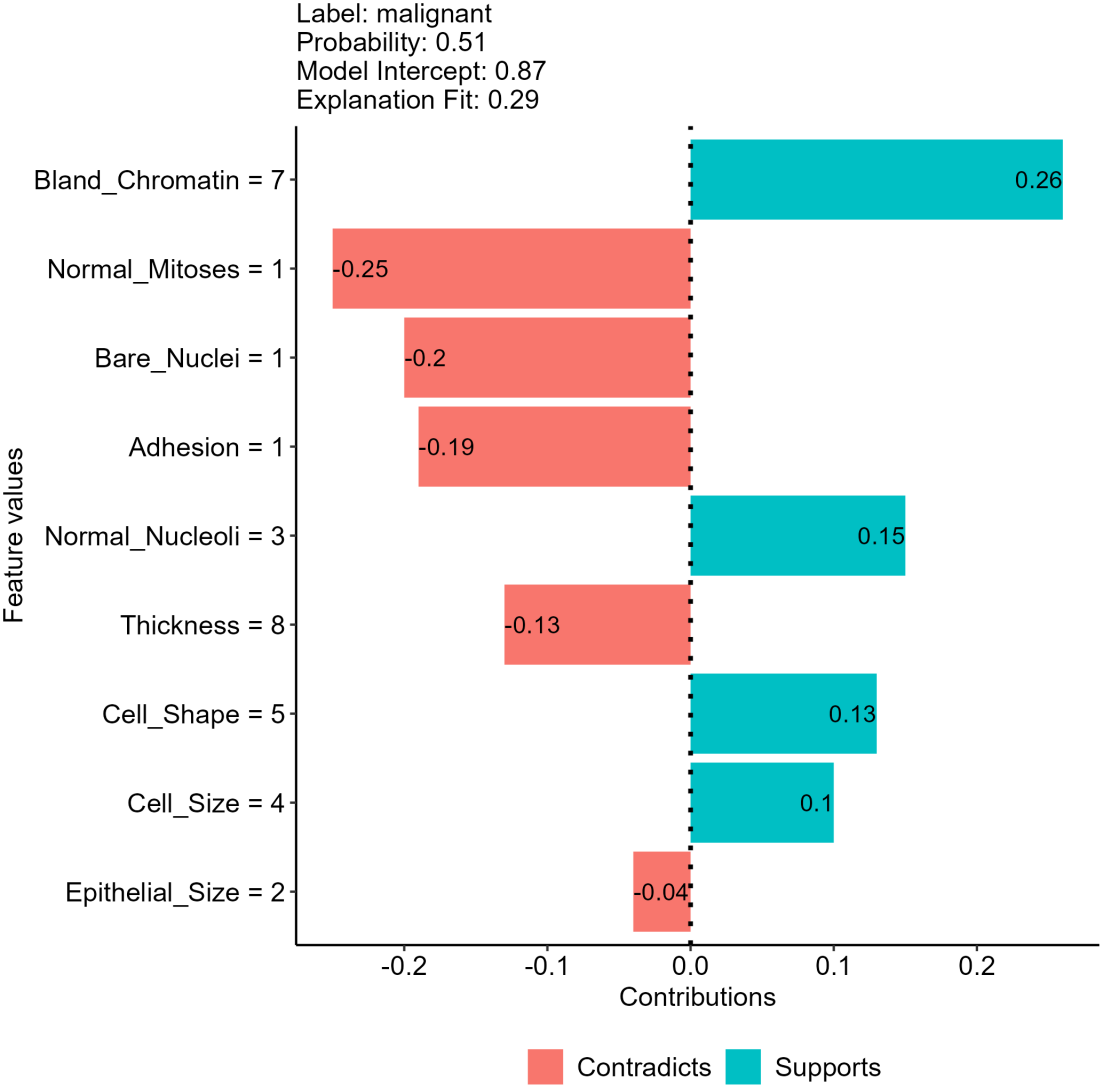
Feature effects on neural network (NNET) model prediction for the breast mass sample randomly selected using a local surrogate model approach.

Another approach to decompose individual predictions is to create an interpretable model (such as a linear or decision tree model) as a surrogate of our model and approximate model behavior by investigating the surrogate model (12, 22). We direct interested readers to the original paper for a detailed description of creating surrogate models (8). **Figure 7** shows the result of using a linear surrogate model to reveal why our NNET model assigns the malignant class to the breast mass sample we selected. Since it is a linear surrogate model, we can visualize feature effects using the coefficients determined by the surrogate model for the sample. For instance, the surrogate model estimated that the bland chromatin score of this sample increased the predicted likelihood of being a malignant tumor by 0.26. This approach focuses on decomposing individual predictions, and thus the surrogate model can be used to investigate feature effects for the mass sample we used to create the model. For other samples, we will need to create other surrogate models using their own data for interpretation.

In addition to the tabular data we have shown, the approach is also useful in interpreting models using text and image data, allowing easy interpretation for models using any data types and algorithms (22, 26). Nevertheless, the approach has several unsolved issues in surrogate model creation processes, such as the methods adopted to select training data and determine the weights of each training data point. The results generated by surrogate models created may vary for the same individual prediction due to the use of different data perturbation, feature selection and weighting methods (11, 22, 26).

##### 3.2.2.3 SHapely additive exPlanations

**Figure 8:**
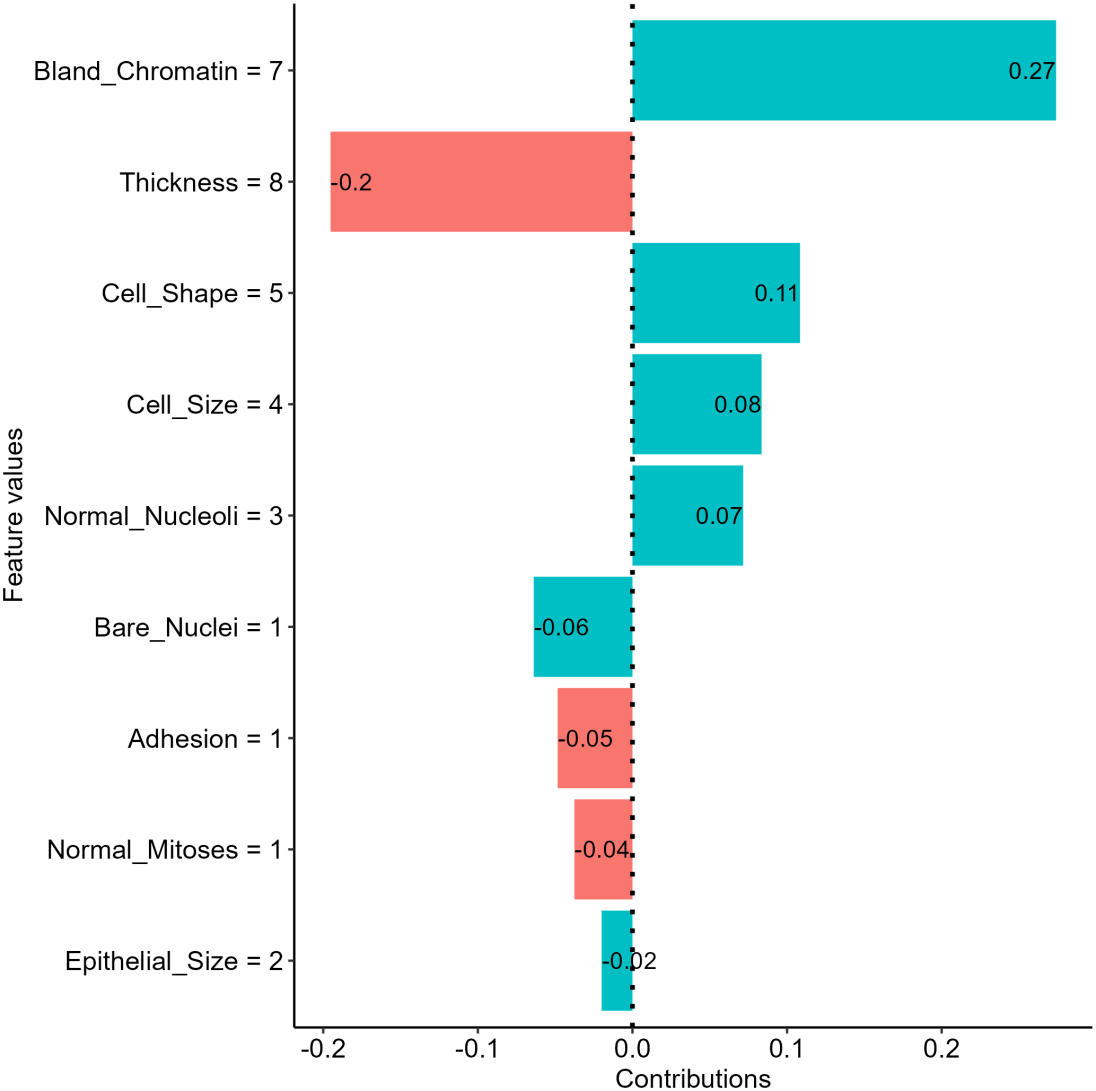
Insights into feature effects on model predictions from the Shapley additive explanation (SHAP) analysis.

The SHapely additive exPlanations (SHAP) approach is another tool that provides local interpretation to drive additional insights into feature effects on individual predictions of black-box ML models. The approach used Shapley values from cooperative game theory addressing how to fairly distribute contributions to players cooperatively finishing a game. In the scenario of ML, features are players, and contributions are the differences in model predictions between the instance of interest and other instances with similar characteristics. Thus, SHAP values are useful in approximating feature contributions to individual predictions of a black-box model. The intuition of the approach is detailed in the original publications (9). In short, the approach used a permutation process similar to the break down plots, while the SHAP approach takes mean probability differences across many or all possible orderings as outputs to avoid the ordering issue (58). Using the SHAP approach, we examined the feature effects on the NNET model prediction for our breast mass example and revealed that the bland chromatin score and thickness score for the sample is the most salient positive and negative contributors, respectively (**Figure 8**).

The approach has gained popularity in the past few years and is suggested for deriving additional insights into feature effects in health literature using ML (32, 60). The major limitations of the approach include computational expense for a large model with many features, the requirement of the training dataset to enable the permutation process, and the inclusion of unrealistic data during the premutation process (58).

##### 3.2.2.4 Ceteris-Paribus plot

**Figure 9:**
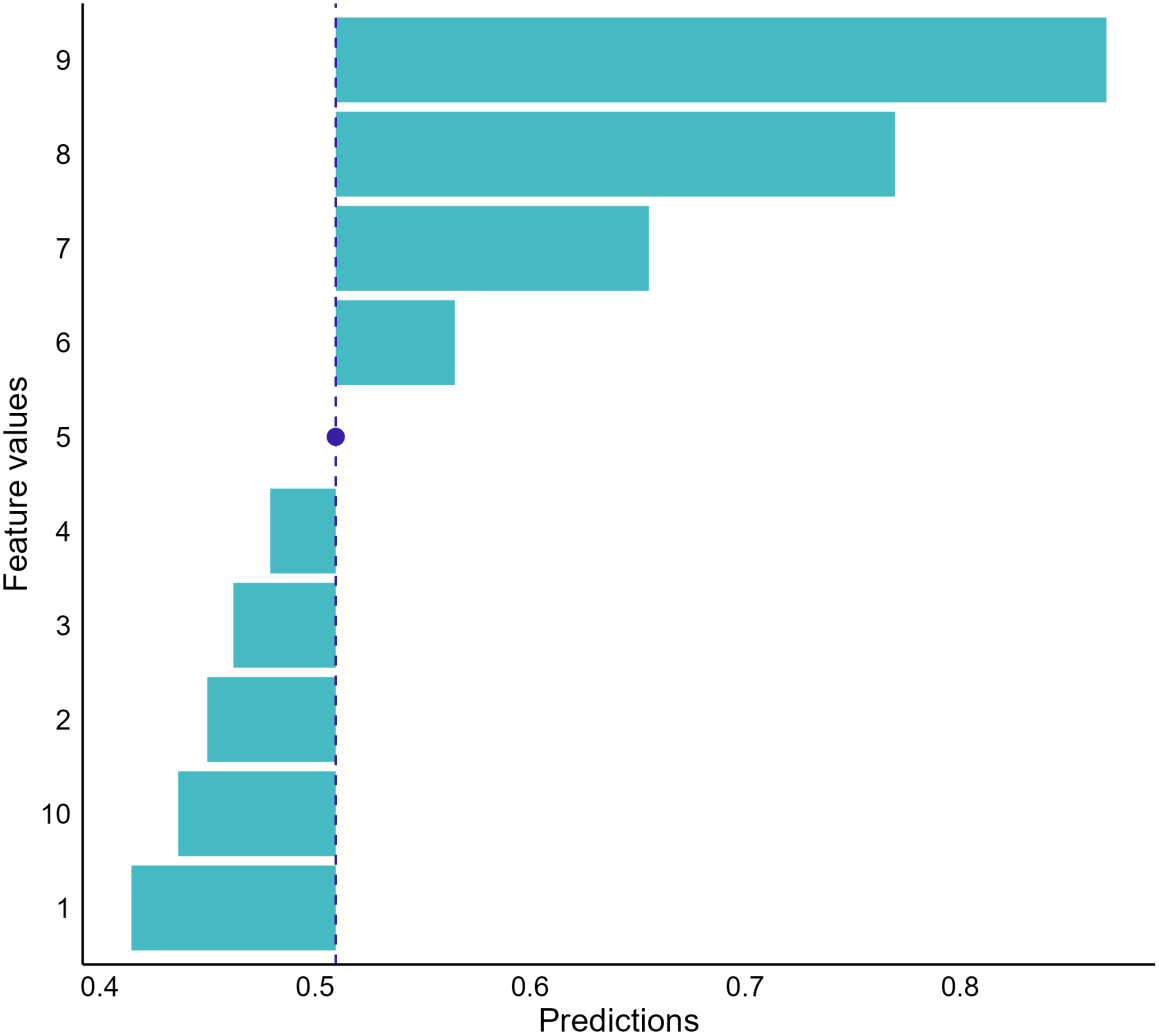
Conditional dependence between cell shape and the neural network prediction for our random-selected breast mass sample. The graph indicates that the cancerous probabilities increase if the sample’s cell shape score increases. The blue dot represents the observed score of cell shape for the sample we selected and the corresponding model prediction.

The last local interpretation approach we covered is Ceteris-Paribus (CP) plots, also named individual conditional expectations (ICE), that address “what-if” questions to provide insights into individual model predictions (61, 62). The approach evaluates the effect of a feature on model predictions by calculating prediction changes when replacing the value of the feature with values of all other features fixed (58). For instance, if we want to examine the dependence between cell shape scores and the NNET model output for the breast mass we selected, we can have the model make predictions on a set of samples with each having a possible score for cell shape and other features with the same value of the selected breast mass sample. Then, we can visualize the predictions to investigate how changes in cell shape scores influence model outputs (**Figure 9**).

The CP plots provide a counterfactual interpretation to quantify feature effects and offer clear visualization to investigate the relationships between model responses and features (62). However, the approach is limited to displaying information for one feature at a time. When the feature number is large, using the approach to decompose model predictions becomes overwhelming because many plots need to be drawn and interpreted. In addition, the approach also assumes no interactions among features (58). Therefore, unrealistic data could be included by the approach to provide misleading information when feature interactions exist.

## 4 Discussion

The black-box model consideration remains one of the biggest challenges to clinical implementation of ML-based tools to inform clinical decisions for oncology care (60, 63–65). As a fast-emerging field, researchers have developed many interpretation approaches deconstructing model predictions from varying aspects to provide additional insights into model predictions. In this manuscript, we provide introductions to various model interpretation techniques, including model-specific, model-agnostic global, and model-agnostic local interpretations, with accompanying examples showing the information these approaches offer along with their respective advantage and disadvantages. Each interpretation provides different insights regarding model behaviors and the effects of the input features. We suggest using these techniques to provide additional insights beyond simple model outputs will help future ML studies in the oncology field translate to the clinic through improved interpretability.

Oncology patients are vulnerable and require carefully planned treatments. Oncologists are often more reluctant to take suggestions without explanations on how the suggestions were generated, resulting in low adoption of the ML-based decision support tools in the field (66, 67). Use of the model interpretations enables information concerning model decision-making processes beyond model outputs. Providing oncologists with this information accompanying model suggestions may be the key to increasing their adoption and enabling the full potential capability of ML models to enhance oncology care (32). Although future research is needed to reveal the impacts of such information on patient care and outcomes, we encourage the use of model interpretation approaches in research and implementation work to examine model decisions, explore their impacts on model development and care practices, and drive novel insights from data into future research.

Various model interpretation techniques are available, and each has its own advantages, disadvantage, and use cases. For instance, model-specific interpretations provide intuitive interpretations by revealing actual model structure, while the utilization of the approaches is limited to models using specific ML algorithms (22, 26). On the other hand, model-agnostic approaches can be applied to any ML models, including ensemble models using multiple ML algorithms, to facilitate the decomposition of varying ML models in the same way to enable comparison between models (26). However, appropriate selection of the approaches to use can be challenging and depends on the characteristics of the datasets used for model training and validation. Use of inappropriate approaches, such as applying PDP to a dataset containing intercorrelated features, can generate misleading information that is not easy to distinguish and may result in unintentional harm (68). Unfortunately, there is no guideline or standard guiding the use of these approaches, however, increasing the awareness of these techniques in the oncology community is an important initial step to establishing the interdisciplinary collaboration involving clinical experts, data scientists, and ML engineers that will lead to more robust interpretation.

Model interpretations, including both model-specific and -agnostic approaches, offer additional benefits beyond uncovering model behaviors by allowing us opportunities to detect biased data and quality issues in our data for model improvement (63). For instance, the interpretations can detect a feature value leading to a certain model decision that is contradictory to clinical knowledge, indicating potential data issues. In a previous analysis using SHAP to explore decision-making processes of models by our team, we showed that alcohol use could protect patients using immune checkpoint inhibitors from short-term readmission [manuscript in press]. This could be a manifestation of reporting bias and data granularity issues instead of related to alcohol consumption. People can be self-selecting in reporting their drinking status and do not always disclose alcohol use, especially heavy use. There could be different levels of use among alcohol users. Light alcohol use may have benefits, while heavy use is obviously harmful. In our case, we used a binary feature to represent alcohol use status that might not be enough to reveal the true effects of alcohol consumption and reduce the discrimination of our models.

Model-agnostic interpretations are capable of enabling additional insights into data health assessment as they are fully data-driven approaches. Data drifting, defined as variations in data used for model development from the data used for model validation and enabling the model after deployment, is a concept that has been increasingly discussed in the ML literature (69, 70). A key factor leading to data variations is time. The meaning, measurement, or definition of a feature enabling the functioning of a model can change over time and result in degradation in model performance or even outright malfunction. For instance, the definition of a disease can change in a short period of time with new evidence discovered. This is particularly true as new markers and therapeutics emerge in the healthcare industry, especially for the oncology area (70). A model may become irrelevant whenever data drifts and continue to provide outputs without any realization of the change in inputs. Use of model-agnostic approaches allows us to detect model dysfunctions by revealing a significant change in model decision-making processes before and after data drift (71).

Despite many advantages, a few general limitations exist across the interpretation approaches in addition to the disadvantages discussed in the previous sections. Model interpretations are not detached from model performance. Misleading information can be a result of interpreting under- or over-fitted models (63, 68). Therefore, we suggest prioritizing model generalizability and applying the interpretation approaches to those high-performing models for additional insights. For model-agnostic approaches, they are incapable of depicting models’ underlying mechanisms of how they process input data to generate decisions. An argument is that the approaches only uncover certain aspects of models that are human-intelligible and leave other parts still in a black box (63). Further, most model-agnostic approaches provide no information on their fidelity to the original models and do not quantify uncertainty generated during the resampling and perturbation (51, 63, 68).

Although there is growing awareness of the need, research in interpretability ML is still in its infant stage and requires more attention. Misuse of the interpretation approaches is likely and can result in unpleasant consequences (68, 72). One future effort can be the development of guidelines for researchers to select approaches suitable to their models and data. Moreover, to our knowledge, these approaches were used mainly in model development and in-silo validation, and less attention was on the impacts of this additional information on care practices and patient outcomes. Increasing in awareness of model interpretability is an essential first step to enabling interdisciplinary approaches for the development and implementation of robust, interpretable ML models (11). Future prospective studies may be feasible to enable thorough suggestions concerning applications and utilizations of the approaches.

## 5 Conclusions

Many ML applications have been developed to support oncology care, but the adoption of the tools among oncologists is low due to challenges in model performance and reproducibility across settings. Introducing interpretability of models can inform poor performance and data quality issues that in turn can be helpful in model development and implementation. In this paper, we provide an accessible introduction to the ideas, use cases, advantages, and limitations of several commonly used model interpretation approaches. We encourage the use of various model-agnostic approaches in ML work supporting oncology care to derive enriched insights from clinical data and report models alongside additional model decision-making process information to allow model utilization and adoption appraisal. Further investigations on the impacts and communication of the model interpretations are needed to enable better utilization of the approaches.

## Author contributions

Conception and design: CS-G, S-CL, Collection and assembly of data: S-CL; Data analysis and interpretation: CS-G, S-CL, CS, CC, DJ. All authors contributed to the article and approved the submitted version.fonc.2023.1129380

## References

[1] Nardini C. Machine learning in oncology: A review. Ecancermedicalscience (2020) 14:1065. doi: 10.3332/ECANCER.2020.1065 32728381 PMC7373638

[2] Melstrom LG Rodin AS Rossi LA Fu P Fong Y Sun V. Patient generated health data and electronic health record integration in oncologic surgery: A call for artificial intelligence and machine learning. J Surg Oncol (2021) 123:52–60. doi: 10.1002/JSO.26232 32974930 PMC7945992

[3] Dlamini Z Francies FZ Hull R Marima R. Artificial intelligence (AI) and big data in cancer and precision oncology. Comput Struct Biotechnol J (2020) 18:2300–11. doi: 10.1016/j.csbj.2020.08.019 PMC7490765 32994889

[4] Ramesh S Chokkara S Shen T Major A Volchenboum SL Mayampurath A. Applications of artificial intelligence in pediatric oncology: A systematic review. JCO Clin Cancer Inform (2021) 5:1208–19. doi: 10.1200/CCI.21.00102 PMC8812636 34910588

[5] Balachandran VP Gonen M Smith JJ DeMatteo RP. Nomograms in oncology: More than meets the eye. Lancet Oncol (2015) 16:e173–80. doi: 10.1016/S1470-2045(14)71116-7 PMC4465353 25846097

[6] Pfob A Lu SC Sidey-Gibbons C. Machine learning in medicine: A practical introduction to techniques for data pre-processing, hyperparameter tuning, and model comparison. BMC Med Res Methodol (2022) 22:1–15. doi: 10.1186/s12874-022-01758-8 36319956 PMC9624048

[7] Sidey-Gibbons JAM Sidey-Gibbons CJ. Machine learning in medicine: A practical introduction. BMC Med Res Methodol (2019) 19:1–18. doi: 10.1186/s12874-019-0681-4 30890124 PMC6425557

[8] Ribeiro MT Singh S Guestrin C. (2016). Why should I trust you? Explaining the predictions of any classifier, in: Proceedings of the 22nd ACM SIGKDD International Conference on Knowledge Discovery and Data Mining, New York, NY, USA (San Francisco, CA: Association for Computing Machinery (ACM)). pp. 1135–44. doi: 10.1145/2939672.2939778

[9] Lundberg SM Lee SI. A unified approach to interpreting model predictions. In: Advances in neural information processing systems. CA, USA: Long Beach (2017). p. 4766–75.

[10] Adadi A Berrada M. Peeking inside the black-box: A survey on explainable artificial intelligence (XAI). IEEE Access (2018) 6:52138–60. doi: 10.1109/ACCESS.2018.2870052

[11] Carvalho DV Pereira EM Cardoso JS. Machine learning interpretability: A survey on methods and metrics. Electron (Basel) (2019) 8:832. doi: 10.3390/electronics8080832

[12] Gilpin LH Bau D Yuan BZ Bajwa A Specter M Kagal L. (2019). Explaining explanations: An overview of interpretability of machine learning, in: Proceedings - 2018 IEEE 5th International Conference on Data Science and Advanced Analytics, DSAA 2018, (Turin, Italy: IEEE). pp. 80–9. doi: 10.1109/DSAA.2018.00018

[13] White house office of science and technology policy. blueprint for an AI bill of rights (2022). Available at: https://www.whitehouse.gov/wp-content/uploads/2022/10/Blueprint-for-an-AI-Bill-of-Rights.pdf (Accessed December 8, 2022).

[14] U.S. department of health and human services food and drug administration. clinical decision support software - guidance for industry and food and drug administration staff . Available at: https://www.fda.gov/drugs/guidance- (Accessed December 8, 2022).

[15] IT Governance Privacy Team. EU General data protection regulation (GDPR). In: An implementation and compliance guide, 3rd ed. Itgp, United Kingdom: IT Governance Publishing (2019). doi: 10.2307/J.CTVR7FCWB

[16] Bertsimas D Wiberg H. Machine learning in oncology: Methods, applications, and challenges. JCO Clin Cancer Inform (2020) 4:885–94. doi: 10.1200/cci.20.00072 PMC7608565 33058693

[17] Cuocolo R Caruso M Perillo T Ugga L Petretta M. Machine learning in oncology: A clinical appraisal. Cancer Lett (2020) 481:55–62. doi: 10.1016/j.canlet.2020.03.032 32251707

[18] Parikh RB Hasler JS Zhang Y Liu M Chivers C Ferrell W. Development of machine learning algorithms incorporating electronic health record data, patient-reported outcomes, or both to predict mortality for outpatients with cancer. JCO Clin Cancer Inform (2022) 6:e2200073. doi: 10.1200/CCI.22.00073 36480775 PMC10166444

[19] Lu S-C Xu C Nguyen CH Geng Y Pfob A Sidey-Gibbons C. Machine learning-based short-term mortality prediction models for cancer patients using electronic health record data: A systematic review and critical appraisal. JMIR Med Inform (2022) 10:e33182. doi: 10.2196/33182 35285816 PMC8961346

[20] Nagy M Radakovich N Nazha A. Machine learning in oncology: What should clinicians know? JCO Clin Cancer Inform (2020) 4, 799–810. doi: 10.1200/cci.20.00049 32926637

[21] Yoon CH Torrance R Scheinerman N. Machine learning in medicine: Should the pursuit of enhanced interpretability be abandoned? J Med Ethics (2022) 48:581–5. doi: 10.1136/medethics-2020-107102 PMC9411871 34006600

[22] Linardatos P Papastefanopoulos V Kotsiantis S. Explainable AI: A review of machine learning interpretability methods. Entropy (2020) 23:18. doi: 10.3390/e23010018 33375658 PMC7824368

[23] Madsen A Reddy S Chandar S. Post-hoc interpretability for neural NLP: A survey. ACM Comput Surv (2021) 55(8):1–42. doi: 10.48550/arxiv.2108.04840

[24] Yuan Q Cai T Hong C Du M Johnson BE Lanuti M. Performance of a machine learning algorithm using electronic health record data to identify and estimate survival in a longitudinal cohort of patients with lung cancer. JAMA Netw Open (2021) 4:e2114723–e2114723. doi: 10.1001/jamanetworkopen.2021.14723 34232304 PMC8264641

[25] Bibault JE Giraud P Burgun A. Big data and machine learning in radiation oncology: State of the art and future prospects. Cancer Lett (2016) 382:110–7. doi: 10.1016/J.CANLET.2016.05.033 27241666

[26] Molnar C. Interpretable machine learning: A guide for making black box models explainable. 2nd. Independently published (2022). Available at: https://christophm.github.io/interpretable-ml-book/.

[27] Murdoch WJ Singh C Kumbier K Abbasi-Asl R Yu B. Definitions, methods, and applications in interpretable machine learning. Proc Natl Acad Sci U.S.A. (2019) 116:22071–80. doi: 10.1073/pnas.1900654116 PMC6825274 31619572

[28] Hakkoum H Abnane I Idri A. Interpretability in the medical field: A systematic mapping and review study. Appl Soft Comput (2022) 117:108391. doi: 10.1016/J.ASOC.2021.108391

[29] Dua D. Graff C. (2019). UCI machine learning repository: Mammographic mass data set (Irvine, CA: University of California, School of Information and Computer Science). http://archive.ics.uci.edu/ml

[30] Ferris MC Mangasarian OL. Breast cancer diagnosis *via* linear programming. IEEE Comput Sci Eng (1995) 2:70–1. doi: 10.1109/MCSE.1995.414885

[31] Jansen T Geleijnse G van Maaren M Hendriks MP ten Teije A Moncada-Torres A. Machine learning explainability in breast cancer survival. Pape-Haugaard LB, Lovis C, Madsen IC, et al (eds) Digital Personalized Health and Medicine. IOS Press, pp 307–311 (2020), 307–11. doi: 10.3233/SHTI200172 32570396

[32] Pfob A Mehrara BJ Nelson JA Wilkins EG Pusic AL Sidey-Gibbons C. Towards patient-centered decision-making in breast cancer surgery: Machine learning to predict individual patient-reported outcomes at 1-year follow-up. Ann Surg (2021) 277:e144–52. doi: 10.1097/SLA.0000000000004862 PMC9762704 33914464

[33] Li R Shinde A Liu A Glaser S Lyou Y Yuh B. Machine learning–based interpretation and visualization of nonlinear interactions in prostate cancer survival. JCO Clin Cancer Inform (2020) 4, 637–46. doi: 10.1200/cci.20.00002 32673068

[34] Hassan AM Lu S-C Asaad M Liu J Offodile ACII Sidey-Gibbons C. Novel machine learning approach for the prediction of hernia recurrence, surgical complication, and 30-day readmission after abdominal wall reconstruction. J Am Coll Surg (2022) 234:918–27. doi: 10.1097/XCS.0000000000000141 35426406

[35] R Core Team. R: A language and environment for statistical computing (2022). Available at: https://www.r-project.org/.

[36] Kuhn M. Caret: Classification and regression training (2022). Available at: https://cran.r-project.org/package=caret.

[37] Biecek P. DALEX: Explainers for complex predictive models in R. J Mach Learn Res (2018) 19:1–5. doi: 10.48550/arXiv.1806.08915

[38] Hvitfeldt E Pedersen TL Benesty M. Lime: Local interpretable model-agnostic explanations (2022). Available at: https://cran.r-project.org/package=lime.

[39] Kobylińska K Mikołajczyk T Adamek M Orłowski T Biecek P. Explainable machine learning for modeling of early postoperative mortality in lung cancer. In: 7th joint workshop on knowledge representation for health care and process-oriented information systems in health care, KR4HC/ProHealth 2019 and the 1st workshop on transparent, explainable and affective AI in medical systems, TEAAM 2019 held in conjuncti (Poznan, Poland: Springer, Cham), vol. 11979. (2019). p. 161–74. doi: 10.1007/978-3-030-37446-4_13

[40] Bertsimas D Dunn J Pawlowski C Silberholz J Weinstein A Zhuo YD. Applied informatics decision support tool for mortality predictions in patients with cancer. JCO Clin Cancer Inform (2018) 2:1–11. doi: 10.1200/CCI.18.00003 PMC6874054 30652575

[41] U.S. Department of Health and Human Services Food and Drug Administration. Good machine learning practice for medical device development: Guiding principles (2021). Available at: https://www.fda.gov/medical-devices/software-medical-device-samd/good-machine-learning-practice-medical-device-development-guiding-principles (Accessed December 8, 2022).

[42] Demir E. A decision support tool for predicting patients at risk of readmission: A comparison of classification trees, logistic regression, generalized additive models, and multivariate adaptive regression splines. Decision Sci (2014) 45:849–80. doi: 10.1111/DECI.12094

[43] Friedman JH Roosen CB. An introduction to multivariate adaptive regression splines. Stat Methods Med Res (1995) 4:197–217. doi: 10.1177/096228029500400303 8548103

[44] Boye KS Lage MJ Shinde S Thieu V Bae JP. Trends in HbA1c and body mass index among individuals with type 2 diabetes: Evidence from a US database 2012–2019. Diabetes Ther (2021) 12:2077. doi: 10.1007/S13300-021-01084-0 34076849 PMC8266935

[45] Jiang T Gradus JL Rosellini AJ. Supervised machine learning: A brief primer. Behav Ther (2020) 51:675–87. doi: 10.1016/J.BETH.2020.05.002 PMC7431677 32800297

[46] Zhang SS Li C Zhang SS Zhang H Pang L Lam K. Using the K-nearest neighbor algorithm for the classification of lymph node metastasis in gastric cancer. Comput Math Methods Med (2012) 2012:11. doi: 10.1155/2012/876545 PMC3488413 23150740

[47] Palczewska A Palczewski J Marchese Robinson R Neagu D. Interpreting random forest classification models using a feature contribution method. In: Bouabana-Tebibel, T., Rubin, S. (eds) Integration of Reusable Systems. Advances in Intelligent Systems and Computing (2014) (Springer: Cham) 263, 193–218. doi: 10.1007/978-3-319-04717-1_9

[48] Polato M Aiolli F. Boolean kernels for rule based interpretation of support vector machines. Neurocomputing (2019) 342:113–24. doi: 10.1016/J.NEUCOM.2018.11.094

[49] Montavon G Samek W Müller KR. Methods for interpreting and understanding deep neural networks. Digital Signal Processing: A Rev J (2018) 73:1–15. doi: 10.1016/J.DSP.2017.10.011

[50] Hayashi Y Setiono R Yoshida K. A comparison between two neural network rule extraction techniques for the diagnosis of hepatobiliary disorders. Artif Intell Med (2000) 20:205–16. doi: 10.1016/S0933-3657(00)00064-6 10998587

[51] Molnar C Casalicchio G Bischl B. Interpretable machine learning – a brief history, state-of-the-art and challenges. Commun Comput Inf Sci (2020) 1323:417–31. doi: 10.1007/978-3-030-65965-3_28

[52] Casalicchio G Molnar C Bischl B. Visualizing the feature importance for black box models. In: Berlingerio M Bonchi F Gärtner T Hurley N Ifrim G, editors. Machine learning and knowledge discovery in databases-European conference, ECML PKDD 2018. Dublin, Ireland: Springer Verlag (2019). p. 655–70. doi: 10.1007/978-3-030-10925-7_40

[53] Fisher A Rudin C Dominici F. All models are wrong, but many are useful: Learning a variable’s importance by studying an entire class of prediction models simultaneously. J Mach Learn Res (2019) 20:1–81. doi: 10.48550/arXiv.1801.01489 PMC8323609 34335110

[54] Iivanainen S Ekstrom J Virtanen H v. KV Koivunen JP. Electronic patient-reported outcomes and machine learning in predicting immune-related adverse events of immune checkpoint inhibitor therapies. BMC Med Inform Decis Mak (2021) 21:205. doi: 10.1186/s12911-021-01564-0 34193140 PMC8243435

[55] Friedman JH. Greedy function approximation: A gradient boosting machine(2001) (Accessed October 18, 2022).

[56] Zhao Q Hastie T. Causal interpretations of black-box models. J Business Economic Stat (2021) 39:272–81. doi: 10.1080/07350015.2019.1624293 PMC7597863 33132490

[57] Apley DW Zhu J. Visualizing the effects of predictor variables in black box supervised learning models. J R Stat Soc Ser B Stat Methodol (2020) 82:1059–86. doi: 10.1111/RSSB.12377

[58] Biecek P Burzykowski T. Explanatory model analysis (2021). New York: Chapman and Hall/CRC. Available at: https://ema.drwhy.ai/ (Accessed October 17, 2022).

[59] Staniak M Biecek P. Explanations of model predictions with live and breakDown packages. R J (2019) 10:395–409. doi: 10.32614/RJ-2018-072

[60] Li R Shinde A Liu A Glaser S Lyou Y Yuh B. Machine learning-based interpretation and visualization of nonlinear interactions in prostate cancer survival. JCO Clin Cancer Inform (2020) 4:637–46. doi: 10.1200/cci.20.00002 32673068

[61] Goldstein A Kapelner A Bleich J Pitkin E. Peeking inside the black box: visualizing statistical learning with plots of individual conditional expectation. J Comput Graphical Stat (2015) 24:44–65. doi: 10.1080/10618600.2014.907095

[62] Lee K Ayyasamy MV. Ji Y Balachandran PV. A comparison of explainable artificial intelligence methods in the phase classification of multi-principal element alloys. Sci Rep (2022) 12:1–15. doi: 10.1038/s41598-022-15618-4 35804179 PMC9270422

[63] Rudin C. Stop explaining black box machine learning models for high stakes decisions and use interpretable models instead. Nat Mach Intell (2019) 1:206–15. doi: 10.1038/s42256-019-0048-x PMC9122117 35603010

[64] Shaw J Rudzicz F Jamieson T Goldfarb A. Artificial intelligence and the implementation challenge. J Med Internet Res (2019) 21:e13659. doi: 10.2196/13659 31293245 PMC6652121

[65] Montani S Striani M. Artificial intelligence in clinical decision support: A focused literature survey. Yearb Med Inform (2019) 28:120–7. doi: 10.1055/s-0039-1677911 PMC6697510 31419824

[66] Kang J Schwartz R Flickinger J Beriwal S. Machine learning approaches for predicting radiation therapy outcomes: A clinician’s perspective. Int J Radiat Oncol Biol Phys (2015) 93:1127–35. doi: 10.1016/j.ijrobp.2015.07.2286 26581149

[67] Kelly CJ Karthikesalingam A Suleyman M Corrado G King D. Key challenges for delivering clinical impact with artificial intelligence. BMC Med (2019) 17:195. doi: 10.1186/s12916-019-1426-2 31665002 PMC6821018

[68] Molnar C König G Herbinger J Freiesleben T Dandl S Scholbeck CA. General pitfalls of model-agnostic interpretation methods for machine learning models. In: Holzinger A Goebel R Fong R Moon T Müller K-R Samek W, editors. xxAI - beyond explainable AI: International workshop, held in conjunction with ICML 2020, July 18, 2020, Vienna, Austria, revised and extended papers. Cham: Springer International Publishing (2022). p. 39–68. doi: 10.1007/978-3-031-04083-2_4

[69] Lu J Liu A Dong F Gu F Gama J Zhang G. Learning under concept drift: A review. IEEE Trans Knowl Data Eng (2019) 31:2346–63. doi: 10.1109/TKDE.2018.2876857

[70] Finlayson SG Subbaswamy A Singh K Bowers J Kupke A Zittrain J. The clinician and dataset shift in artificial intelligence. New Engl J Med (2021) 385:283–6. doi: 10.1056/nejmc2104626 PMC8665481 34260843

[71] Duckworth C Chmiel FP Burns DK Zlatev ZD White NM Daniels TWV. Using explainable machine learning to characterise data drift and detect emergent health risks for emergency department admissions during COVID-19. Sci Rep (2021) 11:1–10. doi: 10.1038/s41598-021-02481-y 34837021 PMC8626460

[72] Slack D Hilgard S Jia E Singh S Lakkaraju H. (2020). Fooling LIME and SHAP: Adversarial attacks on post hoc explanation methods, in: AIES ‘20: Proceedings of the AAAI/ACM Conference on AI, Ethics, and Society, New York, NY (New York, NY: Association for Computing Machinery (ACM)). pp. 180–6. doi: 10.1145/3375627.3375830

